# Okadaic Acid, a Bioactive Fatty Acid from *Halichondria okadai*, Stimulates Lipolysis in Rat Adipocytes: The Pivotal Role of Perilipin Translocation

**DOI:** 10.1155/2013/545739

**Published:** 2013-11-10

**Authors:** Nen-Chung Chang, Aming Chor-Ming Lin, Cheng-Chen Hsu, Jung-Sheng Liu, Leo Tsui, Chien-Yuan Chen, Thanasekaran Jayakumar, Tsorng-Harn Fong

**Affiliations:** ^1^Division of Cardiovascular, Department of Internal Medicine, School of Medicine, College of Medicine, Taipei Medical University Hospital, Taipei, Taiwan; ^2^Department of Emergency, Shin Kong Wu Ho-Su Memorial Hospital, Taipei 11101, Taiwan; ^3^Department of Anatomy, School of Medicine, College of Medicine, Taipei Medical University, No. 250, Wu-Hsing Street, Taipei 11031, Taiwan; ^4^Graduate Institute of Medical Sciences, College of Medicine, Taipei Medical University, Taipei, Taiwan; ^5^Department of Pharmacology, School of Medicine, College of Medicine, Taipei Medical University, Taipei, Taiwan

## Abstract

Lipid metabolism in visceral fat cells is correlated with metabolic syndrome and cardiovascular diseases. Okadaic-acid, a 38-carbon fatty acid isolated from the black sponge *Halichondria okadai*, can stimulate lipolysis by promoting the phosphorylation of several proteins in adipocytes. However, the mechanism of okadaic acid-induced lipolysis and the effects of okadaic acid on lipid-droplet-associated proteins (perilipins and beta-actin) remain unclear. We isolated adipocytes from rat epididymal fat pads and treated them with isoproterenol and/or okadaic acid to estimate lipolysis by measuring glycerol release. Incubating adipocytes with okadaic acid stimulated time-dependent lipolysis. Lipid-droplet-associated perilipins and beta-actin were analyzed by immunoblotting and immunofluorescence, and the association of perilipin A and B was found to be decreased in response to isoproterenol or okadaic acid treatment. Moreover, okadaic-acid treatment could enhance isoproterenol-mediated lipolysis, whereas treatment of several inhibitors such as KT-5720 (PKA inhibitor), calphostin C (PKC inhibitor), or KT-5823 (PKG inhibitor) did not attenuate okadaic-acid-induced lipolysis. By contrast, vanadyl acetylacetonate (tyrosine phosphatase inhibitor) blocked okadaic-acid-dependent lipolysis. These results suggest that okadaic acid induces the phosphorylation and detachment of lipid-droplet-associated perilipin A and B from the lipid droplet surface and thereby leads to accelerated lipolysis.

## 1. Introduction

Adipose tissues play critical roles in energy storage, lipid metabolism, and glucose homeostasis. The development of obesity may be due to excess adipose tissues accumulation or abnormal lipid metabolism. Obesity increases the risk of cardiovascular diseases and diabetes mellitus type 2, comorbidities often detected in metabolic syndrome [1], and visceral fat is linked to metabolic disorders and increased risk of cardiovascular disease [2, 3]. Many hormones and drugs have been identified to participate in the regulation of lipid metabolism, and various hormones and drugs lead to lipolysis through distinct lipolytic pathways [4]. The most studied lipolytic pathway is the cAMP-dependent protein kinase A (PKA) pathway in adipocytes, in which catecholamines bind to beta-adrenoreceptors and activate membrane-bound adenylyl cyclases and thereby increase intracellular cAMP formation [5]. Subsequently, the elevated cAMP levels enhance PKA activity, leading to the phosphorylation and activation of hormone-sensitive lipase (HSL) [6] and lipid-droplet-associated perilipins [7]. Activated HSL and perilipins elicit the hydrolysis of triacylglycerol stored in lipid droplets and the release of free fatty acids and glycerol from adipocytes [8].

Okadaic acid, a polyether derivative of a 38-carbon fatty acid extracted from the black sponge *Halichondria okadai*, is a potent inhibitor of protein phosphatases-1 and -2A (PP1 and PP2A) [9, 10] and a known tumor promoter [11]. Okadaic acid has been used to study various cellular processes such as the cell cycle [12], apoptosis [13], nitric oxide metabolism [14], and calcium signaling [15]. Okadaic acid can stimulate protein phosphorylation rapidly and induce a variety of metabolic processes in diverse cell types [16, 17]. Treating adipocytes with okadaic acid markedly increases the phosphorylation of many proteins and stimulates basal lipolysis [16]. Okadaic acid has been reported to stimulate lipolysis by inducing the translocation of phosphorylated and activated HSL from the cytosol to the lipid-droplets to accelerate lipolysis [18].

In addition to catalytic lipases, perilipins, a family of proteins that coat the surface of lipid droplets [7, 19], have been proposed to regulate lipid metabolism in adipocytes [8, 20]. Perilipin is produced as distinct isoforms that are generated through differential splicing. In adipocytes, perilipin A is the major isoform and perilipin B is the less abundant and shorter variant. Both proteins arise from alternative splicing of mRNA transcripts and share a common N-terminal domain [21]. Phosphorylation of perilipin A is required for the translocation of HSL during PKA-stimulated lipolysis [22]. In adipocytes from perilipin-null animals, basal lipolysis level is higher and stimulated lipolysis is lower than in adipocytes from wild-type mice. These findings suggest that perilipins serve as gatekeeper proteins that are involved in regulating lipid storage and protecting lipids against the hydrolysis by lipases. Phosphorylated perilipins facilitate lipase function following lipolytic stimulation [20]. Okadaic acid treatment can promote perilipin phosphorylation in adipocytes [23], but the underlying molecular mechanism remains unclear. Previously, we showed that globular beta-actin was associated with intracellular lipid droplets in adipocytes [24] and examined how okadaic acid affected lipid-droplet-associated beta-actin.

The clinical implications of okadaic acid have been well documented as okadaic acid administered orally to rats causes intestinal damage, diarrhea, and death but has no detectable effect on the liver [25]. On the other hand, it has been reported that intravenous administration of okadaic acid causes little effect on the intestinal function but severely affects the liver [26]. Apart from these acute effects, the okadaic acid group of toxins seems to have some important chronic effects. These toxins have been found to be potent tumor promoters [27], and the possibility that they are also tumor inducers has been suggested [28]. Although existing observations of human populations are not conclusive, there are epidemiologic lines of evidence that associate these toxins with digestive cancer [29]. 

 In this study, we used okadaic acid to induce lipolysis in primary cultures of isolated rat adipocytes. We report that glycerol release was increased in a time-dependent manner after incubation of cells with okadaic acid. We also analyzed lipid-droplet-associated perilipin A/B and beta-actin by immunoblotting and immunofluorescent labeling and found that treatment with okadaic acid induced the detachment of perilipin A and B but not of beta-actin from the surface of lipid droplets. Furthermore, using specific inhibitors of protein kinase A (KT-5720), protein kinase G (KT-5823), protein kinase C (calphostin C), or tyrosine phosphatases (vanadyl acetylacetonate), we examined the signaling pathways through which okadaic acid induces lipolysis in primary rat adipocytes.

## 2. Materials and Methods

### 2.1. Reagents

Okadaic acid (≥90%), isoproterenol, collagenase (type II), poly-L-lysine, leupeptin, benzamidine, sodium fluoride (NaF), ethylenediaminetetraacetic acid (EDTA), beta-mercaptoethanol, Triton X-100, Tween-20, dimethyl sulfoxide (DMSO), Free Glycerol Determination Kit, diaminobenzidine (DAB), n-propyl gallate, KT-5720, KT-5823, calphostin C, vanadyl acetylacetonate, bovine serum albumin (BSA), tris[hydroxymethyl]aminomethane (Tris), N-tris[hydroxymethyl]methyl-2-aminoethanesulfonic acid (TES), 3-(4,5-dimethylthiazol-2-yl)-2,5-diphenyltetrazolium bromide (MTT), glutaraldehyde, rabbit polyclonal anti-perilipin A/B antibodies, mouse monoclonal anti-beta-actin antibody, and FITC-conjugated anti-rabbit IgG were all purchased from Sigma-Aldrich (St. Louis, MO). Biotin-conjugated anti-rabbit IgG and biotin-conjugated anti-mouse IgG that was preabsorbed with rat serum were purchased from Vector Labs (Burlingame, CA). Streptavidin conjugated with peroxidase was purchased from DAKO (Copenhagen, Denmark). DMEM/F12 medium (phenol-red-free) was purchased from GIBCO (Grand Island, NY), and materials required for sodium dodecyl sulfate-polyacrylamide gel electrophoresis (SDS-PAGE) were purchased from Bio-Rad (Hercules, CA).

### 2.2. Animals

Adult male Wistar rats with body weights in the 200–300 g range were used for the experiments. Rats were housed under a 12:12 h light/dark daily cycle at 23°C and were provided with standard laboratory rat chow and water. All animal care was approved under guidelines established by the Taipei Medical University Ethical Committee for Laboratory Animals.

### 2.3. Preparation and Incubation of Isolated Adipocytes

Rats were injected intraperitoneally with sodium pentobarbital (40 mg/kg body weight) and adipocytes were isolated from the sacrificed rats using procedures described by Rodbell [30] with minor modifications. Briefly, epididymal fat pads were removed immediately after sacrificing animals and were washed and incubated in DMEM/F12 medium (phenol-red-free). The fat pads were minced with scissors and placed in glass vials with medium, and the fat tissue was digested using a collagenase solution (3.3 mg/mL of type-II collagenase in DMEM/F12 medium, pH 7.4, with 3 *μ*M glucose, 4% BSA) with constant shaking at 75 rpm for 30 min at 37°C. Subsequently, fat cells were filtered through a nylon mesh and centrifuged for 5 min at 1000 rpm, after which the supernatant layer of cells was washed thrice with medium to eliminate collagenase. The packed fat cells were resuspended in buffer A (25 mM TES, pH 7.4, 135 mM NaCl, 5 mM KCl, and 1 mM MgCl_2_) at 37°C and adjusted to a density of 1 × 10^5^ cells/mL.

### 2.4. Drug Treatment

Adipocytes were incubated in a total volume of 500 *μ*L in the presence or absence of various reagents (as shown in the figures); 450 *μ*L of fat cells was supplemented with BSA (2.5%, w/v) and incubated with 50 *μ*L of the drug solution (okadaic acid or isoproterenol) for the indicated periods at 37°C in a CO_2_ incubator. The final concentrations of okadaic acid and isoproterenol were 1 *μ*M and 10 *μ*M, respectively. After incubation, the reaction mixture was mixed well and then centrifuged at 1000 rpm for 5 min to separate the medium and the fat cells, which were used to assay for glycerol release and to isolate intracellular lipid droplets, respectively.

### 2.5. Measurement of Lipolysis Based on Glycerol Release

Lipolysis was measured by determining the amount of glycerol released into the medium that was collected after drug treatment and centrifugation; glycerol release was measured using a Free Glycerol Determination Kit. The glycerol content was calculated from absorbance at 540 nm according to the manufacturer's instructions, and glycerol release was expressed as a percentage of the vehicle control (0.1% DMSO).

### 2.6. Isolation of Intracellular Lipid Droplets and SDS-PAGE

Intracellular lipid droplets were isolated from fat cells according to the method of Okuda et al. [31] with minor modification. Briefly, isolated fat cells were washed thrice with normal saline to remove BSA from the reaction medium and then incubated in lysis buffer (5 mM Tris buffer, pH 7.4, 0.025% Triton X-100, 1 mM EDTA, 50 mM NaF, 10 *μ*g/mL leupeptin, and 1 mM benzamidine) on ice for 15 min. The samples were vortexed and then centrifuged at 13000 ×g for 15 min at 4°C. The floating fat-cake fractions were collected and mixed with equal volumes of 2X sample buffer (62.5 mM Tris, pH 6.8, 5% beta-mercaptoethanol, 2% SDS, and 10% glycerol). The samples were heated to 95°C for 5 min and clarified by centrifugation at 10000 rpm for 5 min before using them for SDS-PAGE. Equal amounts of fat-cake extracts were loaded and resolved by SDS-PAGE on 7.5% polyacrylamide slab gels.

### 2.7. Western Blotting

After electrophoresis, proteins were transferred to nitrocellulose membranes and blocked with 5% nonfat milk in Tris-buffered saline (TBS; 50 mM Tris and 200 mM NaCl, pH 7.5), containing 0.05% Tween-20 at room temperature for 1 h and then incubated overnight at 4°C with rabbit polyclonal anti-perilipin A/B antibodies or mouse monoclonal anti-beta-actin antibody diluted in TBS. After washing with TBS, the membrane strips were incubated with biotin-conjugated anti-rabbit IgG or biotin-conjugated anti-mouse IgG (rat serum preabsorbed to avoid nonspecific binding between mouse and rat) at room temperature for 1 h, washed with TBS, and then incubated with peroxidase-conjugated streptavidin at room temperature for 1 h to enhance the signals of immunoreactive bands. After washing again with TBS, immunoreactive bands were visualized by exposing membranes to a diaminobenzidine solution (5% diaminobenzidine and 0.02% H_2_O_2_ in TBS).

### 2.8. Immunofluorescent Labeling of Isolated Intracellular Lipid Droplets

Isolated intracellular lipid droplets were placed on 10% poly-L-lysine-coated slides for 20 min at room temperature for adhesion. After washing with phosphate-buffered saline (PBS: 137 mM NaCl, 2.7 mM KCl, 1.5 mM KH_2_PO_4_, and 8 mM Na_2_HPO_4_, pH 7.4), the isolated lipid droplets were fixed using 2% glutaraldehyde in PBS for 5 min. After washing with PBS, the lipid droplets were incubated with 5% non-fat milk in PBS for 30 min at room temperature to block nonspecific binding sites. The samples were next incubated with rabbit polyclonal anti-perilipin A/B antibodies at 4°C overnight. After washing with PBS, the samples were incubated with FITC-conjugated anti-rabbit IgG for 1 h at room temperature. After washing again with PBS, the samples were mounted with 2% n-propyl gallate and 60% glycerol in PBS (pH 8.0), sealed in place using nail polish, and examined using a Nikon epifluorescence microscope (Nikon, Tokyo, Japan).

### 2.9. Statistical Analysis

Data are presented as the mean ± SE from at least 3 independent experiments. The significance of differences between experimental and control groups was assessed using Student's *t*-test; *P* < 0.05 was considered statistically significant.

## 3. Results

### 3.1. Okadaic Acid Induces Lipolysis in a Time-Dependent Manner

**Figure 1:**
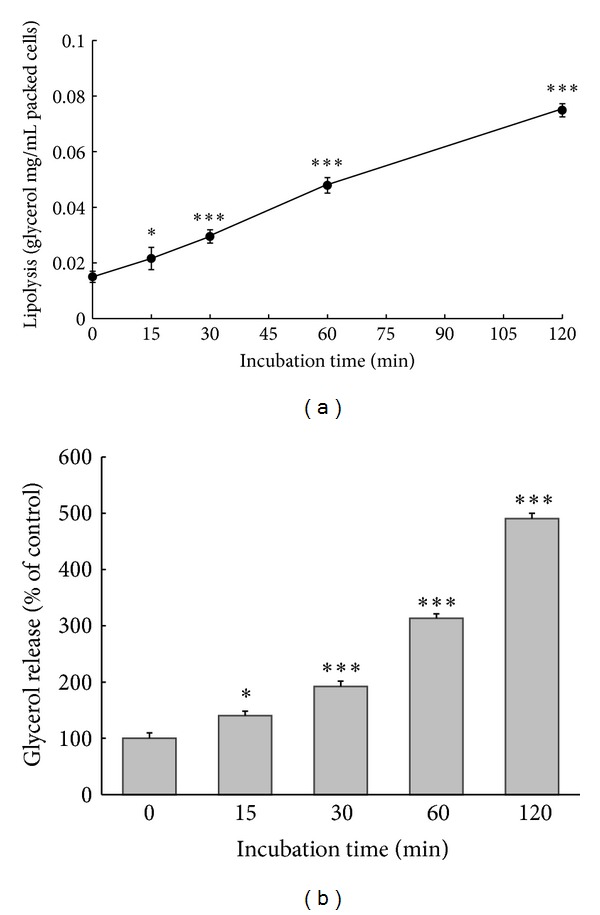
Time course of okadaic-acid-induced glycerol release in rat adipocytes. Fat cells were incubated with okadaic acid (1 *μ*M, 37°C) for the indicated periods. After incubation, media were collected and glycerol release was measured as described in Materials and Methods. Glycerol released into the media was assayed as an index of lipolysis and expressed as mg/mL of packed cell volume (a). Glycerol release data are expressed as percentages of the values obtained after incubation for 0 min (b). Each point represents the mean ± SE of three separate experiments. *P < 0.05 and ***P < 0.001 compared with values of 0 min incubation.

 We investigated the effect of okadaic acid on lipolysis in rat visceral fat cells. Okadaic acid (1 *μ*M) increased glycerol release by cultured adipocytes in a time-dependent manner (Figure 1(a)): the amounts of glycerol released were 15.5  ±  0.2, 21.6 ± 0.8, 29.5 ± 0.2, 47.9 ± 0.1, and 74.9 ± 2.3 *μ*g/mL of packed cells following treatment with okadaic acid for 0, 15, 30, 60, and 120 min, respectively. During incubation for 15, 30, 60, and 120 min, lipolysis increased 1.4-, 1.9-, 3.1-, and 4.8-fold, respectively (Figure 1(b)).

### 3.2. Okadaic Acid Treatment Diminishes the Association of Perilipins A and B with Lipid Droplets

**Figure 2:**
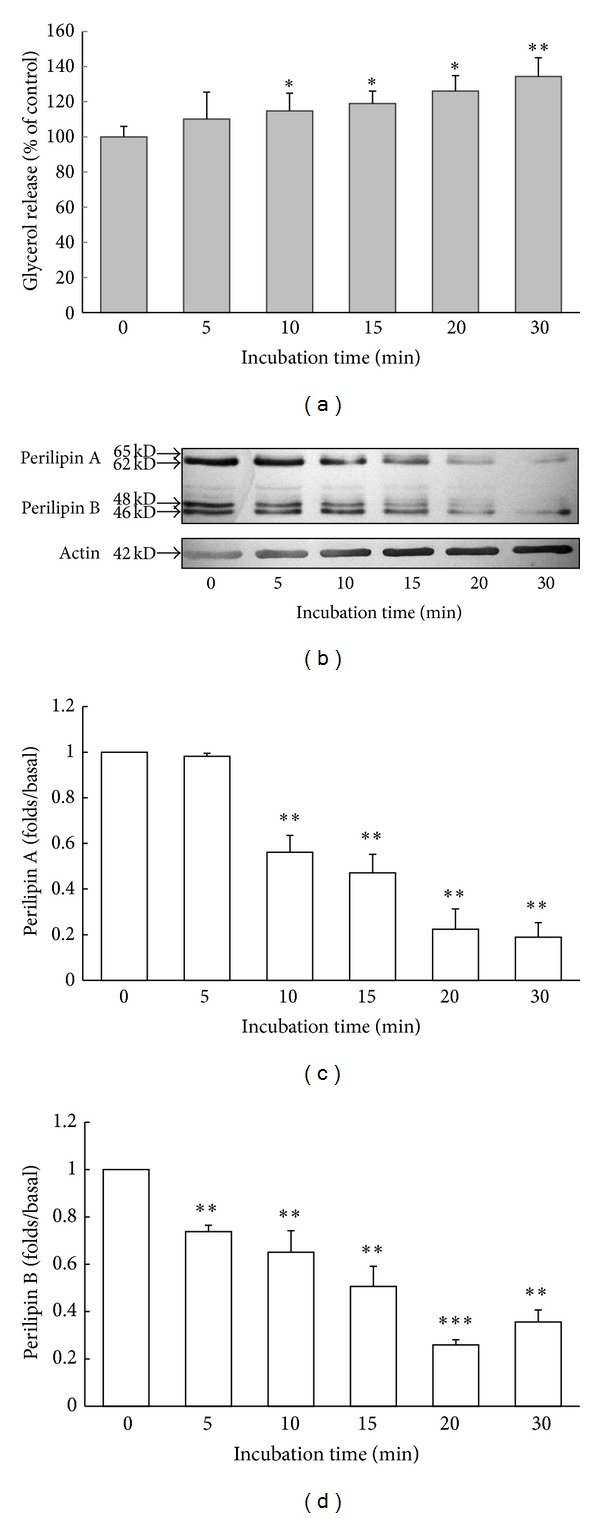
Effects of okadaic acid on lipid-droplet-associated perilipin A and B in rat adipocytes. Cells were incubated with okadaic acid (1 *μ*M, 37°C) for the indicated periods. After incubation, each reaction mixture was centrifuged to separate the medium from the fat cells. Glycerol release was measured as described in Materials and Methods, and glycerol release data are expressed as percentages of the values obtained after incubation for 0 min (a). After incubation, fat cells were homogenized and centrifuged and the proteins of the fat cake fraction were separated using SDS-PAGE (7.5% gels) and subjected to immunoblotting with a primary antibody against perilipins. Immunoblots showing perilipin A (62 kD and 65 kD) and B (46 kD and 48 kD) or beta-actin (42 kD) from okadaic-acid-treated cells (b). Immunoreactive perilipin A (c) and perilipin B (d) were quantified as a percentage relative to the density in unstimulated fat cells. Each point represents the mean ± SE of three separate experiments; *P < 0.05, **P < 0.01, and ***P < 0.001 compared with values of 0 min incubation. The shift in the molecular weight of perilipin A from 62 kD to 65 kD could be observed after 5 min stimulation with okadaic acid.

To explore the role of lipid-droplet-associated perilipins in okadaic-acid-induced lipolysis, we used immunoblotting to examine perilipins during the 120 min treatment with okadaic acid. Almost no lipid-droplet-associated perilipin A was detected after cells were treated for 30 min with okadaic acid (data not shown), and, therefore, we tested shorter incubation times. To investigate the short-term effects of okadaic acid on lipolysis, fat cells were treated with 1 *μ*M okadaic acid for 0, 5, 10, 15, 20, and 30 min. Glycerol release increased only slightly after incubation with okadaic acid for 5 min but increased substantially after incubation for 10, 15, 20, and 30 min (Figure 2(a)). Furthermore, we used immunoblotting to examine lipid-droplet-associated perilipins during the 30 min incubation with okadaic acid. The rabbit polyclonal anti-perilipin A/B antibodies can label native perilipin A (62 kD), phosphorylated perilipin A (65 kD), and perilipin B (46 and 48 kD). We detected phosphorylated perilipin A (65 kD) after 5 min treatment with okadaic acid and observed that the levels of lipid-droplet-associated perilipin A (62 kD and 65 kD) and perilipin B (46 kD and 48 kD) decreased gradually following okadaic acid treatment (Figure 2(b)). Quantification of these results demonstrated that total perilipin A decreased only by 0.98-fold after 5 min but decreased significantly after 10, 15, 20, and 30 min incubation by 0.56-, 0.47-, 0.22-, and 0.18-fold, respectively (Figure 2(c)). The levels of total perilipin B decreased significantly after 5, 10, 15, 20, and 30 min incubation by 0.73-, 0.65-, 0.51-, 0.26-, and 0.35-fold, respectively (Figure 2(d)). By contrast, the level of lipid-droplet-associated beta-actin (42 kD) did not decrease noticeably during the 30 min incubation with okadaic acid (Figure 2(b)).

### 3.3. Okadaic Acid and Isoproterenol Induce Detachment of Perilipins from Lipid Droplets

**Figure 3:**
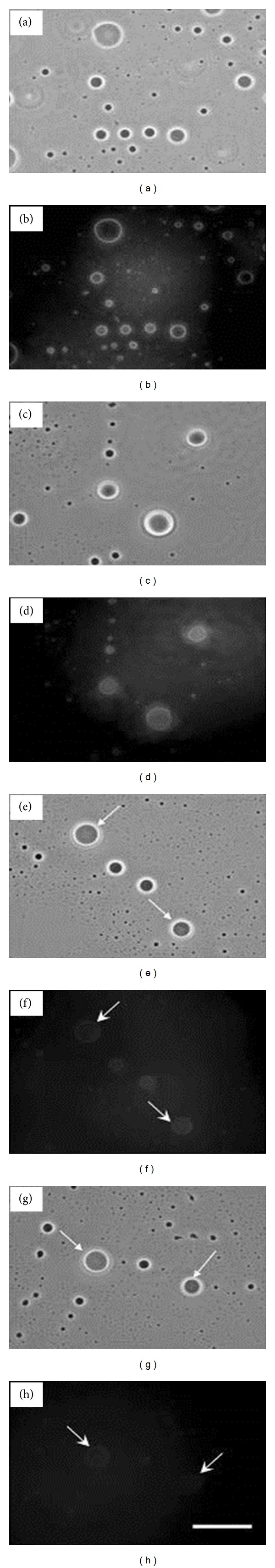
Immunofluorescent labeling of perilipin A/B in isolated intracellular lipid droplets. Adipocytes were incubated with buffer A, DMSO, isoproterenol (10 *μ*M), or okadaic acid (1 *μ*M) for 2 h at 37°C. Perilipins were assembled as a bright ring surrounding the surface of lipid droplets in buffer A control (a and b) and DMSO (c and d), but labeling for perilipins was weak on lipid droplets in the isoproterenol-stimulated group (e and f) and absent on lipid droplets in the okadaic-acid-stimulated group (g and h). Figures (a), (c), (e), and (g) show phase-contrast images of figures (b), (d), (f), and (h), respectively. Arrows indicate isolated intracellular lipid droplets. Bar = 20 *μ*m.

 We used immunofluorescent labeling to investigate morphologically how isoproterenol and okadaic acid affect lipid-droplet-associated perilipins. Labeling with the polyclonal anti-perilipin antibodies revealed bright fluorescence along the circumference of the isolated intracellular lipid droplets in the buffer-A group (Figures 3(a) and 3(b)) and in the 0.1%-DMSO group (Figures 3(c) and 3(d)). This result indicated that perilipins are not only coisolated with intracellular lipid droplets but also associated with the surface of lipid droplets.

When we incubated fat cells with isoproterenol (10 *μ*M) or okadaic acid (1 *μ*M) for 120 min and then isolated intracellular lipid droplets for immunofluorescent labeling, only weak fluorescence was detected surrounding the lipid droplets in the isoproterenol-treated group (Figures 3(e) and 3(f)) and okadaic-acid-treated group (Figures 3(g) and 3(h)). This result suggested that most of the perilipins detached from the surface of lipid droplets after cells were incubated with the drugs.

### 3.4. Okadaic Acid Enhances Isoproterenol-Induced Lipolysis

**Figure 4:**
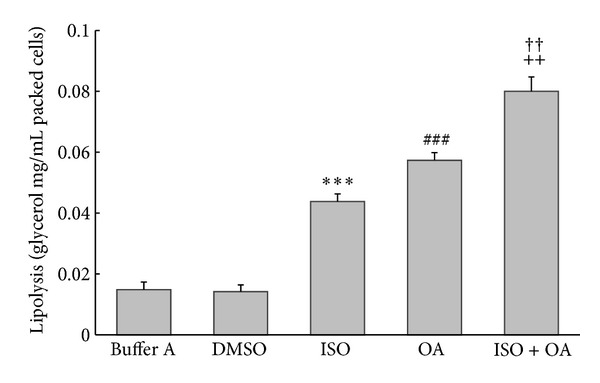
Okadaic acid enhances isoproterenol-induced glycerol release. Adipocytes were incubated for 2 h with isoproterenol (10 *μ*M) or okadaic acid (1 *μ*M) or both. Glycerol release was assayed (in triplicate) as an index of lipolysis and expressed as mg/mL of packed cell volume. Data are the mean ± SE of three separate experiments. ***P < 0.001 compared with buffer A group, ^###^ *P* < 0.001 compared with DMSO-treated group, ^++^ *P* < 0.01 compared with isoproterenol-treated group, and ^††^ *P* < 0.01 compared with okadaic acid-treated group. ISO: isoproterenol; OA: okadaic acid.

Isoproterenol (10 *μ*M) or okadaic acid (1 *μ*M) has been shown previously to induce lipolysis [16]. To compare the signal transduction pathways in the lipolysis induced by okadaic acid and isoproterenol, we combined the 2 drugs and stimulated fat cells. Incubation of cells with isoproterenol (10 *μ*M) for 120 min stimulated glycerol release that was 3.0-fold higher than in the buffer-A group, and incubation with okadaic acid (1 *μ*M) for 120 min stimulated glycerol release that was 4.1-fold higher than in the DMSO group; however, incubation of cells with both isoproterenol and okadaic acid for 120 min stimulated 5.6-fold higher glycerol release compared to the DMSO group (Figure 4). These results demonstrated that okadaic acid treatment can enhance isoproterenol-induced lipolysis in rat adipocytes.

### 3.5. A Tyrosine-Phosphatase Inhibitor but Not Inhibitors of PKA, PKC, and PKG Regulates Okadaic-Acid-Induced Lipolysis

**Figure 5:**
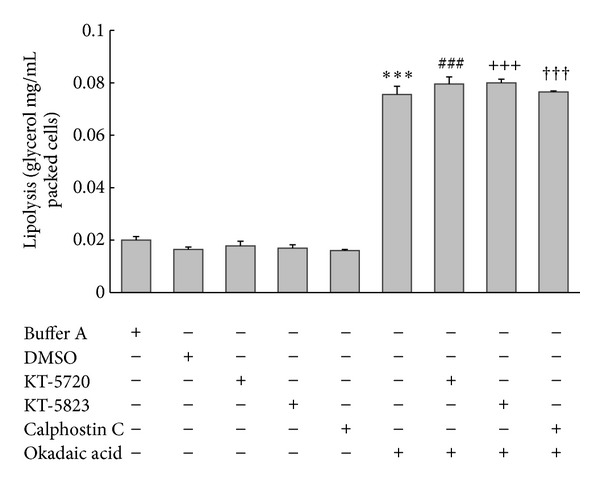
Effects of inhibitors of PKA, PKG, and PKC on okadaic acid-induced lipolysis in rat adipocytes. Cells were incubated with inhibitor alone or okadaic acid (1 *μ*M) in the absence or presence of inhibitors of PKA (KT-5720; 0.3 *μ*M), PKG (KT-5823; 3 *μ*M), or PKC (calphostin C; 0.5 *μ*M) for 2 h at 37°C; media were then collected and glycerol release was measured. ***P < 0.001 compared to the DMSO-treated group, ^###^ *P* < 0.001 compared to the KT-5720-treated group, ^+++^ *P* < 0.001 compared to the KT-5823-treated group, and ^†††^ *P* < 0.001 compared to the calphostin-C-treated group.

**Figure 6:**
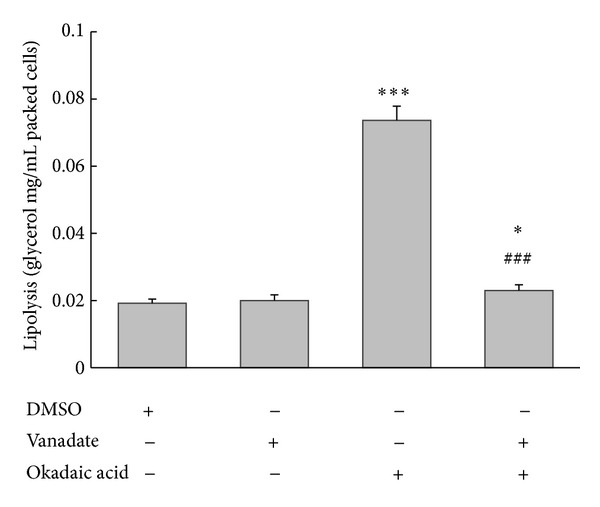
Effect of tyrosine phosphatase inhibition on okadaic-acid-induced lipolysis in rat adipocytes. Cells were incubated with okadaic acid (1 *μ*M) in the absence or presence of vanadyl acetylacetonate (300 *μ*M) for 2 h at 37°C. The medium was collected and glycerol release was determined. *P < 0.01 and ***P < 0.001 compared to the DMSO-treated group, ^###^ *P* < 0.001 compared to the okadaic-acid-treated group. Vanadate: vanadyl acetylacetonate.

To determine whether PKA, PKG, and PKC affect okadaic-acid-induced lipolysis in adipocytes, we used KT-5720 (specific inhibitor of PKA), KT-5823 (specific inhibitor of PKG), and calphostin C (specific inhibitor of PKC). Basal level of glycerol release was not affected by treatment with these 3 inhibitors, and preincubating cells with the inhibitors did not abolish or attenuate okadaic-acid-induced lipolysis (Figure 5). By contrast, preincubation with the tyrosine-phosphatase blocker vanadyl acetylacetonate (300 *μ*M) potently inhibited okadaic-acid-induced lipolysis (Figure 6). The data suggested that tyrosine phosphatases, but not PKA, PKG, or PKC, are involved in okadaic-acid-induced lipolysis in rat visceral fat cells.

## 4. Discussion

Our results have demonstrated that treatment of adipocytes with okadaic acid can induce lipolysis in a time-dependent manner. Perilipin A (62 kD) and B (46 and 48 kD) and beta-actin (42 kD) were abundant in quiescent fat cells and were associated with lipid droplets isolated from these cells. After incubating adipocytes for 5 min with okadaic acid, phosphorylated perilipin A (65 kD) was detected, and following okadaic acid treatment for 10 min, the amounts of perilipin A and B associated with lipid droplets decreased and glycerol release increased substantially. Furthermore, okadaic-acid-induced lipolysis was suppressed by an inhibitor of tyrosine phosphatases but not by inhibitors of PKA, PKG, or PKC. These results suggest that treatment with okadaic acid activates tyrosine phosphatases and leads to perilipin A phosphorylation, which results in the detachment of perilipin A and B from the surface of lipid droplets and leads to lipolysis and glycerol release in rat visceral adipocytes.

Okadaic acid, a polyether derivative of fatty acid, can penetrate the plasma membrane readily and inhibit PP1 and PP2A potently [16, 17]. When adipocytes are incubated with 1 *μ*M okadaic acid, which is sufficient for inhibiting PP1 and PP2A, the phosphorylation of many proteins is increased and glycerol release is stimulated in adipocytes [16, 23]. PP1 and PP2A are abundant in rat adipocytes and also the major phosphatases in these cells [32]. PP2A is the principal phosphatase responsible for dephosphorylating HSL in adipocytes [33]. Conversely, PP1 is the main phosphatase that dephosphorylates perilipin in adipocytes [34]. Thus, treatment with okadaic acid could inhibit both PP1 and PP2A to enhance the phosphorylation of both perilipin and HSL and stimulate lipolysis. In our study, treatment with okadaic acid (1 *μ*M, for 2 h) increased the release of glycerol 4.8-fold in freshly prepared fat cell suspensions (1 × 10^5^ cells/mL). Previously, glycerol release was increased by approximately 11-fold in freshly prepared fat cell suspensions (3 × 10^5^ cells/mL) incubated with 2 *μ*M of okadaic acid for 3 h [35]. Thus, okadaic acid might induce lipolysis in a concentration- and time-dependent manner.

 Lipid-droplet-associated perilipin is considered to act as a barrier or gatekeeper with critical roles in regulating cellular lipid metabolism [8, 20]. Perilipin A (62 kD) is a prominent phosphoprotein that becomes phosphorylated after isoproterenol treatment, after which the protein migrates as a 65/67 kD doublet in SDS-PAGE and is also the most heavily radiolabeled protein in the cell [7]. The anti-perilipin A/B polyclonal antibodies we used in this study can recognize native perilipin A (62 kD), phosphorylated perilipin A (65 kD), and two perilipin B bands (46 and 48 kD) on immunoblots. The 65/67 kD doublet of phosphorylated perilipin A was not identified here possibly because we did not use radiolabels or because of the limitations of the anti-perilipin antibody.

 Treatment with isoproterenol can stimulate the phosphorylation and translocation of perilipin from the lipid-droplet surface to the cytosol in rat adipocytes [36, 37]. We observed here that okadaic acid treatment of rat adipocytes also leads to the detachment of perilipin A and B from the surface of lipid droplets. Thus, phosphorylation may trigger perilipin disassociation from the surface of lipid droplets. Moreover, Morimoto et al. [18] reported that okadaic acid treatment does not increase the catalytic activity of HSL but induces the translocation of HSL to the lipid droplets. Phosphorylated HSL translocates from the cytosol to the surface of lipid droplets [37, 38], and phosphorylated perilipins might detach from the surface of droplets to expose the triacylglycerol stored in lipid droplets and then facilitate lipid hydrolysis by HSL [20, 22].

 On the other hand, we found that when adipocytes were incubated with okadaic acid or isoproterenol, at different incubation periods (0–30 min), the expression levels of beta-actin in lipid droplets remain unchanged. The cytoskeleton proteins associated with lipid droplets seem to be resistant to drug treatment. For example, formation of vimentin intermediate filamentous cages enclosed and protected the nascent lipid droplets of 3T3-L1 adipocytes from disruption of colchicine treatment [39]. Hence, we suggested that the lipid-droplet-associated beta-actin might be a stable structural protein on the surface of intracellular lipid droplet.

 In this study, isoproterenol-induced lipolysis was enhanced by okadaic acid treatment, which agrees with the previous results [16]. Isoproterenol-stimulated lipolysis, but not okadaic-acid-induced lipolysis, was antagonized by insulin in a previous study [35], and we found that okadaic-acid-induced lipolysis was not inhibited by a PKA blocker (KT-5720). These findings suggest that okadaic acid and isoproterenol stimulate lipolysis through distinct signaling pathways.

 PKA is involved in catecholamine- or isoproterenol-induced lipolysis, and PKG is responsible for lipolysis stimulated by atrial natriuretic peptide [4]. Because the specific inhibitors of PKA (KT-5720) and PKG (KT-5823) did not abolish or attenuate okadaic-acid-induced glycerol release in our study, we suggest that neither PKA nor PKG is involved in okadaic-acid-induced lipolysis in rat adipocytes. Okadaic acid has been reported to inhibit PP2A and activate protein kinase B (PKB) [40] and then promote the translocation of glucose transporter 4 (GLUT4) from cytosol to cell membrane and increase glucose uptake in adipocytes [41]. In the case of okadaic acid treatment, glucose uptake and GLUT4 translocation in rat adipocytes and 3T3-L1 preadipocytes might be mediated through the activation of PKC-zeta and/or PKC-lambda [42]. These reports suggest that PKB and PKC are responsible for okadaic-acid-induced glucose uptake. Calphostin C, a specific PKC inhibitor, did not block okadaic-acid-induced lipolysis in this study, indicating that PKC is not directly involved in okadaic-acid-induced lipolysis. Additional experiments are required to determine whether PKB signaling participates in okadaic acid-induced lipolysis.

 In rat adipocytes, vanadate mimics the antidiabetic effects of insulin and also inhibits catecholamine-induced lipolysis [43]. However, the antilipolytic action of vanadate is distinct mechanistically from that of insulin because wortmannin, a phosphoinositol 3-kinase (PI3K) inhibitor that blocked the antilipolytic effect of insulin, failed to block vanadate-mediated antilipolysis [35], indicating that PI3K did not participate in antilipolytic signaling initiated by vanadate. Vanadate has been shown to inhibit protein tyrosine phosphatases (PTPases) [44] and activate membranous nonreceptor protein tyrosine kinases (membPTKs) and thereby stimulate protein tyrosine phosphorylation in rat adipocytes [45]. It is reported that several organic vanadyl compounds had anti-lipolytic activity. The vanadyl acetylacetonate had the most powerful potency, and it correlated better with the inhibition of adipose membrane-PTPase in cell-free experiments [35]. Moreover, the PTK blocker staurosporine can reverse the antilipolytic effect of vanadate [45]. These findings suggest that the inhibitory effect of vanadyl acetylacetonate on okadaic-acid-induced lipolysis might be mediated by PTPases or membPTKs in rat adipocytes. However, the effects of PTPases and membPTKs on HSL and perilipins warrant further investigation.

 In conclusion, we found that one treatment with okadaic acid resulted in detachment of perilipin A and B from lipid droplets. The molecular weight of perilipin A shifted from 62 kD to 65 kD after incubating cells with okadaic acid for 5 min, and after perilipins detached from lipid droplets, glycerol release increased. Although okadaic acid and isoproterenol might produce their lipolytic effects through distinct mechanisms in rat adipocytes, both drugs induced the detachment of perilipins from lipid droplets and stimulated glycerol release. This study suggested that okadaic acid may regulate lipid metabolism by mediating the activity of protein phosphatases and the translocation of perilipins.
